# Topical Application of *Aronia melanocarpa* Extract Rich in Chlorogenic Acid and Rutin Reduces UVB-Induced Skin Damage via Attenuating Collagen Disruption in Mice

**DOI:** 10.3390/molecules25194577

**Published:** 2020-10-07

**Authors:** Young Her, Tae-Kyeong Lee, Jong Dai Kim, Bora Kim, Hyejin Sim, Jae-Chul Lee, Ji Hyeon Ahn, Joon Ha Park, Ji-Won Lee, Junkee Hong, Sung-Su Kim, Moo-Ho Won

**Affiliations:** 1 Department of Dermatology, Kangwon National University Hospital, Kangwon National University School of Medicine, Chuncheon, Gangwon 24289, Korea; youngderma@knuh.or.kr; 2 Department of Biomedical Science and Research Institute for Bioscience and Biotechnology, Hallym University, Chuncheon, Gangwon 24252, Korea; tk-lee@hallym.ac.kr; 3 Division of Food Biotechnology, School of Biotechnology, Kangwon National University, Chuncheon, Gangwon 24341, Korea; jongdai@kangwon.ac.kr; 4 Department of Neurobiology, School of Medicine, Kangwon National University, Chuncheon, Gangwon 24341, Korea; nbrkim17@gmail.com (B.K.); janny20@naver.com (H.S.); anajclee@kangwon.ac.kr (J.-C.L.); jh-ahn@ysu.ac.kr (J.H.A.); 5 Department of Physical Therapy, College of Health Science, Youngsan University, Yangsan, Gyeongnam 50510, Korea; 6 Department of Anatomy, College of Korean Medicine, Dongguk University, Gyeongju, Gyeongbuk 38066, Korea; jh-park@dongguk.ac.kr; 7 Famenity Co., Ltd., Uiwang, Gyeonggi 16006, Korea; jiwon.lee@famenity.com (J.-W.L.); jk.hong@famenity.com (J.H.)

**Keywords:** *Aronia melanocarpa*, collagens, epidermal thickness, matrix metalloproteinases, skin photodamage

## Abstract

*Aronia melanocarpa*, a black chokeberry, contains high levels of phenolic acids and polyphenolic flavonoids and displays antioxidative and anti-inflammatory effects. Through high-performance liquid chromatography for extracts from *Aronia melanocarpa*, we discovered that the extract contained chlorogenic acid and rutin as major ingredients. In this study, we examined the protective effects of the extract against ultraviolet B- (UVB)-induced photodamage in the dorsal skin of institute of cancer research (ICR) mice. Their dorsal skin was exposed to UVB, thereafter; the extract was topically applied once a day for seven days. Photoprotective properties of the extract in the dorsal skin were investigated by clinical skin severity score for skin injury, hematoxylin and eosin staining for histopathology, Masson’s trichrome staining for collagens. In addition, we examined change in collagen type I and III, and matrix metalloproteinase (MMP)-1 and MMP-3 by immunohistochemistry. In the UVB-exposed mice treated with the extract, UVB-induced epidermal damage was significantly ameliorated, showing that epidermal thickness was moderated. In these mice, immunoreactivities of collagen type I and III were significantly increased, whereas immunoreactivities of MMP-1 and 3 were significantly decreased compared with those in the UVB-exposed mice. These results indicate that treatment with *Aronia melanocarpa* extract attenuates UV-induced photodamage by attenuating UVB-induced collagen disruption: these findings might be a result of the chlorogenic acid and rutin contained in the extract. Based on the current results, we suggest that *Aronia melanocarpa* can be a useful material for developing photoprotective adjuvant.

## 1. Introduction

Exposure to ultraviolet (UVB) radiation induces skin damage including erythema, excoriation, dryness, and crust formation [1,2]. UVB radiation causes photodamage primarily through reactive oxygen species (ROS) produced in the epidermis [3,4]. ROS induces secretion of matrix metalloproteinases (MMPs; MMP-1, -2, -3, -9, and -13) from fibroblasts and keratinocytes, which in turn degrade collagen and other extracellular matrix (ECM) proteins, impair collagen synthesis, and cause skin photodamage [5]. Many studies have shown that antioxidant-rich natural extracts block UV-stimulated MMP production through dermal fibroblasts [6].

*Aronia melanocarpa*, a black chokeberry, has been used to treat cardiovascular diseases, to lower blood pressure, and to reduce blood glucose level through its antioxidant activity [7,8]. In addition, it exhibits anti-inflammatory, gastroprotective, antidiabetic, and hepatoprotective properties [9,10].

*Aronia melanocarpa* contains a large amount of biologically active components including polyphenolic flavonoids (flavonols, anthocyanins, flavan-3-ols, etc.) and phenolic acids [11,12,13,14]. In addition, *Aronia melanocarpa* is rich in anthocyanins and proanthocyanidins, which exhibit strong antioxidant properties, reduce the risks of certain lifestyle diseases and inhibit aging [15,16]. Furthermore, Denev et al. (2019) recently reported that chlorogenic acid and rutin were present in *Aronia melanocarpa* [17]. Chlorogenic acid, as an ester of L-quinic acid and caffeic acid, has been studied to bring beneficial physiological properties including antioxidant effects [18,19,20]. Rutin (3-rhamnosyl-glucosylquercetin) is a flavonol glycoside and has been reported to display antioxidant and anti-inflammatory efficacies [19,21,22].

Lately, sunblock products are in great request for protection against UVB exposure, and the radioprotective effects of numerous materials including natural resources have been studied [4,23,24]. However, effects on skin aging and photodamage of *Aronia melanocarpa* have been poorly reported. Therefore, we here explore the protective efficacy of extract from *Aronia melanocarpa* in terms of histopathological change, change in collagens, which are main structural proteins in the extracellular matrix of diverse connective tissues in the body, and MMPs, which are capable of degrading all kinds of extracellular matrix proteins, in mouse dorsal skin after exposure to UVB irradiation.

## 2. Results

### 2.1. Ingredients of Aronia Melanocarpa Extract (AME)

**Figure 1:**
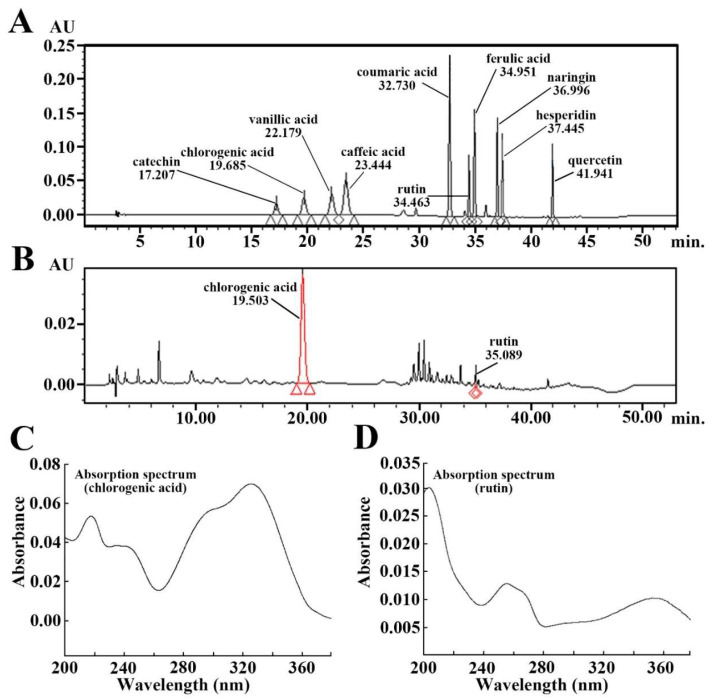
(**A** and **B**) HPLC chromatograms of standard phenolic compounds (**A**) and aronia melanocarpa extract (AME) (**B**). The retention times of the standard phenolic compounds are 17.207 min (catechin), 19.685 min (chlorogenic acid), 22.179 min (vanillic acid), 23.444 min (caffeic acid), 32.730 min (coumaric acid), 34.463 min (rutin), 34.951 min (ferulic acid), 36.996 min (naringin), 37.445 min (hesperidin) and 41.941 min (quercetin). The retention times of the AME are 19.503 min (chlorogenic acid) and 35.089 min (rutin). (**C** and **D**) The absorption spectrum of chlorogenic acid (**C**) and rutin (**D**).

As shown in Figure 1A, the standard sample containing phenolic compounds was chromatographed by high-performance liquid chromatography (HPLC), among the phenolic compounds, chlorogenic acid and rutin peaked at 19.685 min and 34.463 min, respectively (Figure 1A). In this study, we found that AME contained chlorogenic acid (19.503 min) and rutin (35.089 min) in the chromatographed AME (Figure 1B). Each absorption spectrum of chlorogenic acid and rutin is displayed in Figure 1C,D.

### 2.2. Clinical Skin Severity (CSS) Score

**Figure 2:**
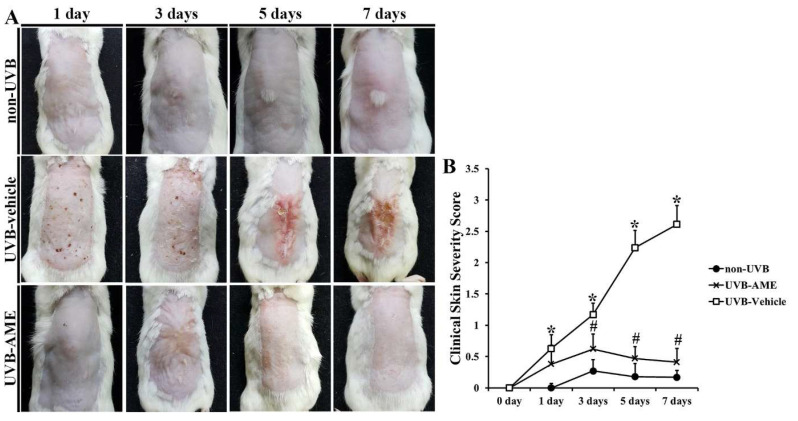
(**A**) Images of the dorsal skin in the non-ultraviolet B -(UVB), UVB-vehicle and UVB-AME groups at 1, 3, 5 and 7 days after UVB irradiation. In the UVB-AME group, the clinical skin severity (CSS) is remarkably attenuated compared to that in the UVB-vehicle group. (**B**) CSS score in each group at 1, 3, 5 and 7 days after UVB irradiation. The CSS score in the UVB-AME group is decreased from 3 days after UVB irradiation. Bars indicate the means ± SEM (*n* = 10 in each group, ^*^ *p* < 0.05 vs. non-UVB group, ^#^ *p* < 0.05 vs. UVB-vehicle group).

Mouse dorsal skin damage due to exposure of UVB irradiation was investigated according to specific indicators (scarring/dryness, edema, erythema/hemorrhage, and excoriation/erosion), which were described in a previous study [2]. In the non-UVB group, the CSS score was not significantly altered until seven days (Figure 2A,B). The CSS score in the UVB-vehicle group was remarkably increased with time after UVB-exposition, showing that the score at seven days after UVB irradiation was 2.61; in particular, the CSS score showed the most drastic increase range at five days after UVB irradiation (Figure 2A,B). In contrast, the CSS score in the UVB-AME group was significantly low compared to that in the UVB-vehicle group; especially, from three days after UVB irradiation, the CSS score in that group started to be lowered compared with that in the UVB-vehicle group. The CSS score at seven days after UVB irradiation was 0.41 (Figure 2A,B).

### 2.3. Epithermal Thickness and Fibroblasts by Hematoxylin and Eosin (H and E) Staining

**Figure 3:**
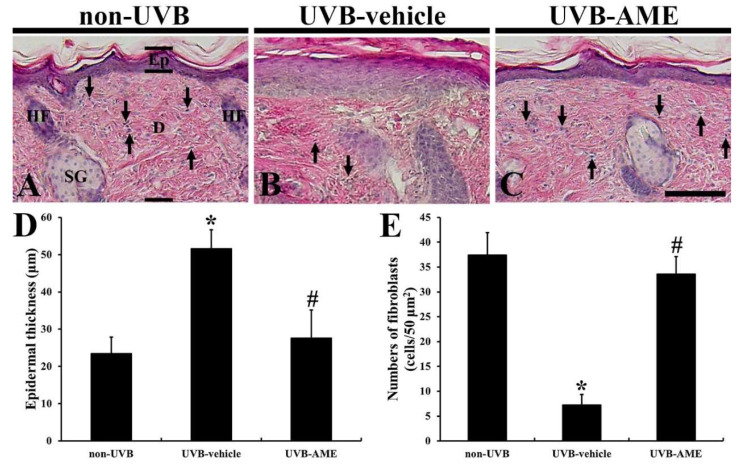
(**A**–**C**) Hematoxylin and eosin (H and E) staining in the dorsal skin of the non-UVB (**A**), UVB-vehicle (**B**) and UVB-AME (**C**) groups at 7 days after UVB irradiation. The epidermis (Ep) is thickened, and numbers of fibroblasts (arrows) are reduced in the non-UVB group. In the UVB-AME group, epidermal thickness and numbers of fibroblasts are apparently recovered. D, dermis; HF, hair follicle; SG, sebaceous gland. Scale bar = 50 μm. (**D** and **E**) Mean epidermal thickness (**D**) and mean numbers of fibroblasts (**E**) in the dorsal skin of the non-UVB, UVB-vehicle and UVB-AME groups at 7 days after UVB irradiation. Bars indicate the means ± SEM (*n* = 10 in each group, ^*^ *p* < 0.05 vs. non-UVE group, ^#^ *p* < 0.05 vs. UVB-vehicle group).

Intact epidermis containing keratinocytes and melanocytes, and dermis containing fibroblasts were clearly shown in the non-UVB group (Figure 3A). In this group, average thickness of the epidermis was 23.4 μm (Figure 3D), and the number of fibroblasts was 37.4 cells/50 μm^2^ (Figure 3E). However, in the UVB-vehicle group, the epidermis was apparently thickened (51.6 μm), showing that the cells in the epidermis were hypertrophied (Figure 3B,D). In addition, fibroblast cells located in the dermis were hypertrophied, and their numbers were significantly reduced (7.2 cells/50 μm^2^) compared to the non-UVB group (Figure 3B,E). In the UVB-AME group, on the other hand, damage in the cells was apparently attenuated compared with that in the non-UVB group (Figure 3C). In this group, the mean epidermal thickness was significantly decreased (27.6 μm), and the number of fibroblast cells were significantly increased (33.6 cells/50 μm^2^) by AME treatment compared to the UVB-vehicle group (Figure 3D,E).

### 2.4. Collagen Fibers by Masson’s Trichrome Staining

**Figure 4:**
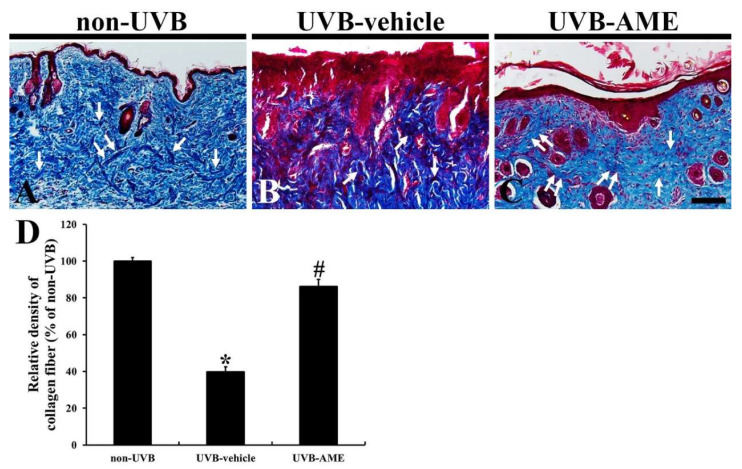
(**A**–**C**) Masson’s trichrome staining in the dorsal skin of the non-UVB (**A**), UVB-vehicle (**B**) and UVB-AME (**C**) at 7 days after UVB irradiation. Thick collagen fibers (arrows) are well stained with aniline blue in the dermis of the non-UVB group. In the UVB-vehicle group, significantly progressed fibrosis with reduction in collagen fibers is shown. However, in the UVB-AME group, the disruption of collagen fibers is attenuated. Scale bar = 100 μm. (**D**) Relative density of collagen fibers in the dorsal skin of each group at 7 days after UVB irradiation. Bars indicate the means ± SEM (*n* = 10 in each group, ^*^ *p* < 0.05 vs. non-UVE group, ^#^ *p* < 0.05 vs. UVB-vehicle group).

In the non-UVB group, collagen fibers were thickly stained with aniline blue in the dermis of mouse dorsal skin and abundantly found (Figure 4A). In the UVB-vehicle group, however, significantly progressed fibrosis induced by UVB irradiation in the dermis was shown, which was stained with Biebrich scarlet-acid (Figure 4B). In this group, collagen fiber density was remarkably reduced (39.8% of the non-UVB group) compared with that in the non-UVB group (Figure 4D). On the other hand, in the UVB-AME group, ameliorated fibrosis was found, showing that the density of collagen fibers was significantly increased (86.2% of the non-UVB group) by AME treatment compared to that in the UVB-vehicle group (Figure 4C,D).

### 2.5. Collagen Fibers by Immunohistochemistry

**Figure 5:**
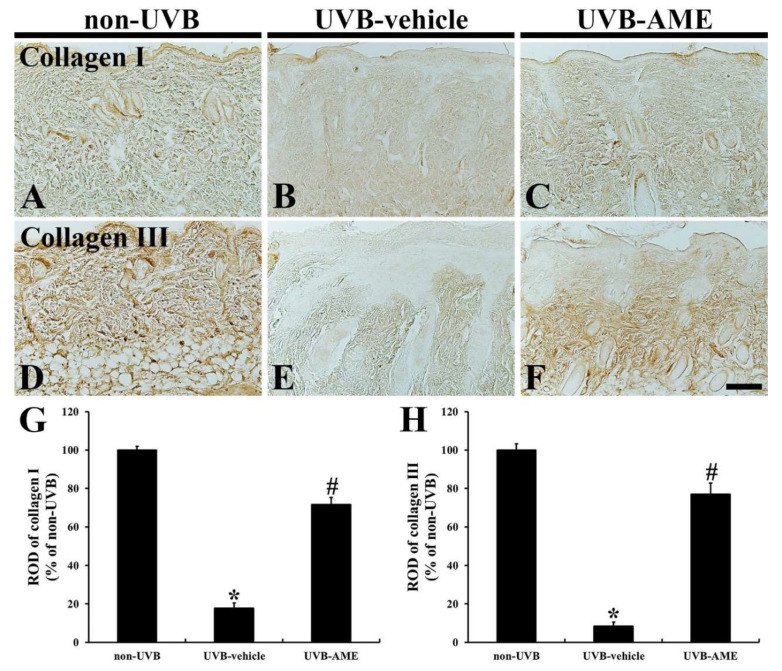
(**A**–**F**) Immunohistochemistry for collagen I (**A**–**C**) and III (**D**–**F**) in the dorsal skin of the non-UVB (**A** and **D**), UVB-vehicle (**B** and **E**) and UVB-AME (**C** and **F**) at 7 days after UVB irradiation. In the UVB-vehicle group, collagen I and III immunoreactivity is apparently diminished compared to that in the non-UVB group. In the UVB-AME group, however, collagen I and III immunoreactivity is significantly reserved. Scale bar = 100 μm. (**G** and **H**) relative optical densities (RODs) of collagen I (**G**) and III (**H**) immunoreactivity in the dorsal skin of each group at 7 days after UVB irradiation. Bars indicate the means ± SEM (*n* = 10 in each group, ^*^ *p* < 0.05 vs. non-UVE group, ^#^ *p* < 0.05 vs. UVB-vehicle group).

Moderate immunoreactivity of type I collagen (collagen I) was shown in the dermis of the dorsal skin of the non-UVB group (Figure 5A). However, collagen I immunoreactivity in the dorsal skin of the UVB-vehicle group was remarkably diminished (17.8% vs. that in the non-UVB group) compared to that in the non-UVB group (Figure 5B,G). On the other hand, in the UVB-AME group, collagen I immunoreactivity in the dermis was significantly strengthened (71.6% vs. that in the non-UVB group) by AME treatment compared with that in the UVB-vehicle group (Figure 5C,G).

Type III collagen (collagen III) immunoreactivity in the dermis of the non-UVB group was strongly and plentifully observed (Figure 5D). In contrast, in the UVB-vehicle group, collagen III immunoreactivity was remarkably reduced (8.4% vs. that in the non-UVB group) compared to that in the non-UVE group (Figure 5E,H). On the other hand, collagen III immunoreactivity in the UVB-AME group was significantly reserved (77.1% vs. that in the non-UVB group) by AME treatment compared to that in the UVB-vehicle group (Figure 5F,H).

### 2.6. MMPs by Immunohistochemistry

**Figure 6:**
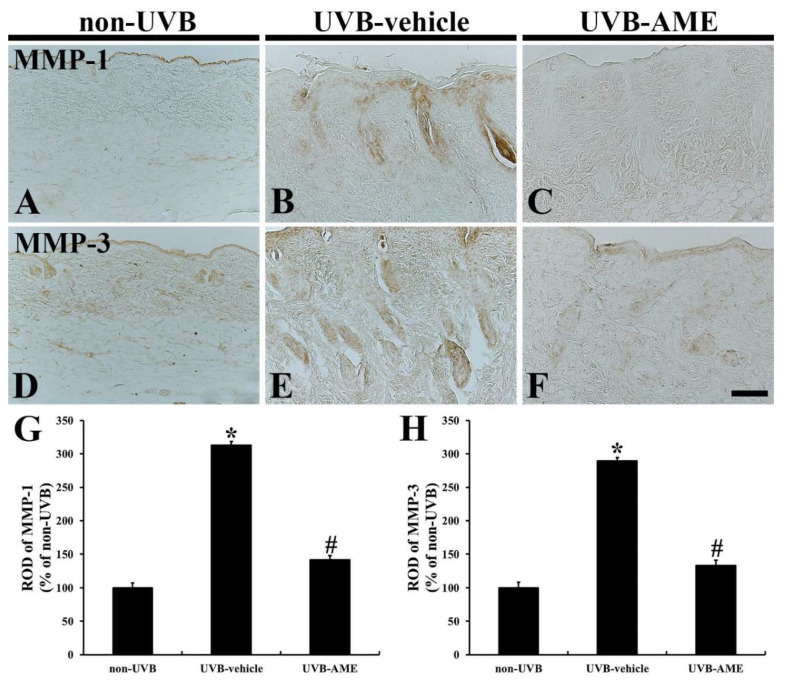
(**A**–**F**) Immunohistochemistry for matrix metalloproteinase-1 (MMP-1) (**A**–**C**) and 3 (**D**–**F**) in the dorsal skin of the non-UVB (**A** and **D**), UVB-vehicle (**B** and **E**) and UVB-AME (**C** and **F**) at 7 days after UVB irradiation. In the UVB-vehicle group, MMP-1 and 3 immunoreactivity is significantly enhanced in the dermis. However, in the UVB-AME group, MMP-1 and 3 immunoreactivity is significantly weakened compared to that in the UVB-vehicle group. Scale bar = 100 μm. (**G** and **H**) RODs of MMP-1 (**G**) and 3 immunoreactivity in the dorsal skin of each group at 7 days after UVB irradiation. Bars indicate the means ± SEM (*n* = 10 in each group, ^*^ *p* < 0.05 vs. non-UVE group, ^#^ *p* < 0.05 vs. UVB-vehicle group).

In the non-UVB group, weak MMP-1immunoreactivity was shown in the dermis of the dorsal skin (Figure 6A). However, in the UVB-vehicle group, MMP-1 immunoreactivity was significantly enhanced (312.8% of the non-UVB group) compared with that in the non-UVB group (Figure 6B,G). In the UVE-AME group, on the other hand, MMP-1 immunoreactivity was significantly reduced (141.7% of the non-UVB group) by AME treatment compared to that in the UVE-vehicle group (Figure 6C,G).

Moderated MMP-3 immunoreactivity was shown in the dorsal skin of the non-UVB group (Figure 6D). In contrast, a markedly increased MMP-3 immunoreactivity (289.3% of the non-UVB group) was found in the UVB-vehicle group compared to that in the non-UVB group (Figure 6E,H). However, in the UVB-AME group, MMP-3 immunoreactivity was significantly decreased (133.7% of the non-UVB group) by AME treatment compared to that in the UVB-vehicle group (Figure 6F,H).

## 3. Discussion

Here, we investigated effects of topical AME on skin damage including changes in MMPs and collagens in mouse dorsal skin following UVB irradiation. Our present results show that UVB-induced gross changes such as thickened epidermis, decreased fibroblasts, disrupted collages and increased MMPs were attenuated by topical application of AME.

Berries of *Aronia melanocarpa* are rich in valuable materials, exhibit a strong antioxidant activity and afford potential therapeutic benefits (hepatoprotective, gastroprotective, antiproliferative and anti-inflammatory activities) [9,10,25,26,27]. Among the activities, the antioxidant activity includes free radical scavenging, changes in effects of transition metals on oxidation, and inhibition of lipid peroxidation in several model systems, showing that AME is rich in bioactive polyphenols such as anthocyanins, flavanols, proanthocyanidins, and phenolic acids [28]. In this study, we detected two phenolic compounds (chlorogenic acid and rutin) from AME through HPLC analysis. Chlorogenic acid has been reported as exerting beneficial effects against UVB-exposed skin damage. In detail, Cha et al. (2014) demonstrated that chlorogenic acid protected human adult low calcium high temperature (HaCaT) keratinocytes from UVB-induced damage via scavenging ROS including superoxide anion and hydroxyl radicals [20]. A precedent study reported that mice fed chlorogenic acid displayed minor dermatitis in their dorsal skin irradiated by UVB compared to that of vehicle-treated mice [29]. Similarly, in our current study, a photoprotective property of the AME was found in the mouse dorsal skin by the CSS score for skin injury.

In this study, damage in the fibroblasts located in the dermis was apparent in the UVB-vehicle group, but the damage was significantly attenuated in the UVB-AME group. This finding is supported by reports that show that rutin, one of the ingredients of AME, protected against photodamage in the human fibroblasts CCD111Sk and human dermal fibroblasts (HDFs) cells via enhancing ROS scavenging activity [22,30]. Additionally, Choi et al. (2014) demonstrated that topical application of rutin onto the dorsal skin prevented UVB-induced inflammatory responses via inhibiting cyclooxygenase-2 (COX-2) and inducible nitric oxide synthase (iNOS) in HR-1 hairless mice [21].

Based on the presented HPLC results, AME contained various kinds of phenolic compounds. Among the compounds, except with the chlorogenic acid and rutin, a peak of the chromatogram for the test sample (retention time, about 41 min) corresponded with that of standard sample showing that the AME contained a trace amount of quercetin. Some precedent studies have described that AME contains quercetin [18,19]. The quercetin belongs to flavonoid group of polyphenols, and its antioxidant efficacy was demonstrated by many studies. For example, Yin et al. (2013) demonstrated that quercetin decreased UVB-induced generation of reactive oxygen species (ROS) in an in vitro model of JB6 P+ mouse epidermal C141 cells. Additionally, in that study, the quercetin protected skin damage induced by UVB irradiation via reducing ROS-induced DNA damage and superoxide anion production and elevating antioxidant enzyme expressions in SKH-1 hairless mice [31]. Moreover, pre-treatment with quercetin conferred neuroprotective effects via increasing antioxidant enzymes including Cu, Zn-superoxide dismutase (SOD1), Mn-superoxide dismutase (SOD2), catalase and glutathione peroxidase in a gerbil model of transient global cerebral ischemia [32].

It is well known that UVB irradiation triggers erythema, cutaneous edema, hyperplasia, leukocyte infiltration, dermal blood vessel dilation and vascular hyperpermeability, and the irradiation, ultimately, causes epidermal proliferation and skin thickening [33]. In particular, collagens are degraded and collagen production is inhibited in the fibroblasts located in the dermis after UV irradiation [34]. In our current study, decreased immunoreactivities of collagen type I and III in the UVB-vehicle group were significantly increased in the UVB-AME group, showing that enhanced immunoreactivities of MMP-1 and 3 were significantly decreased in the UVB-AME group. UVB radiation can evoke DNA damage and overproduction of ROS [10]. The produced ROS activates downstream key transcription factors such as mitogen-activated protein kinase (MAPK) and nuclear factor-κB (NFκB) and nuclear factor-erythroid 2-related factor-2 (Nrf2) and finally triggers the secretion of pro-inflammatory cytokines including interleukin (IL)-1β and tumor necrosis factor-α (TNF-α) [35]. In addition, excessive accumulation of ROS increases MMP activity, resulting in destruction of extracellular matrix (ECM) via collagen degradation [36]. Additionally, it has been reported that topical administration of AME ameliorates pro-inflammatory response via suppression of ROS-MAPK-NF-kB signaling pathway in a mouse model of tetradecanoylphorbol-13-acetate (TPA)-induced ear edema model [37].

We recently reported that topical application of Oenanthe javanica extract (OJE) on mouse dorsal skin protected UVB-induced skin damage via ameliorating collagen disruption and inflammatory responses [2]. However, there was a limitation of the qualitative analysis of the ingredients contained in the OJE; it was poorly acknowledged which components of the OJE contributed to the protective effect against UVB-induced skin damage. In a few studies, AME showed an improvement in inflammation in a mouse model of ear edema [37], but the active ingredient of the extract was not reported. Our present results present not only the protective effect of the AME against UVB-induced skin damage but also present major ingredients of the AME containing phenolic compounds including chlorogenic acid and rutin. In this regard, compared to the precedent studies, our current study provides more optimized background data of the AME as a UVB protective material.

In summary, our present results show that topical application of AME protected skin damage in mouse dorsal skin following UVB irradiation via attenuating collagen disruption. Based on both the previous and current studies, since AME contained chlorogenic acid and rutin, it showed protective effects against UVB-induced skin damage. Therefore, we suggest that AME can be a useful material for developing photoprotective adjuvant.

## 4. Materials and Methods

### 4.1. Experimental Animals

Thirty institute of cancer research (ICR) mice (male, 7 week-old, 33–37 g of body weight) were obtained from the Experimental Animal Center of Kangwon National University (Chuncheon, Republic of Korea). The housing conditions for the animals were optimally established as follows: (1) suitable room temperature (23–26 °C) and relative humidity (50–60%) were maintained, (2) constant dark and light cycle was controlled and (3) freely accessible feed and water were provided. The procedure of the present study was authorized by the Institutional Animal Care and Use Committee (IACUC) at Kangwon National University (approval no., KW-200121-2) and stuck to guidelines from the current international laws and policies in “Guide for the Care and Use of Laboratory Animals” (The National Academies Press, 8th Ed., 2011).

### 4.2. Preparation and Treatment of AME

Fruits of *Aronia melanocarpa* were harvested from Chuncheon (Gangwon, Republic of Korea). They were washed with distilled water and air-dried at 60 °C and crushed into a fine powder via a grinder (IKA M20, IKA, Staufen, Germany). Thereafter, the powder was refluxed with pure water at 70 °C for 12 h. The AME was filtrated via Whatman No. 1 filter paper (Whatman Ltd., Maidstone, Kent, UK) and concentrated with vacuum evaporator at 40 °C. Finally, the AME was completely dried through lyophilization. The extract was stored at −20 °C until used. The extract procedure was conducted and the AME was provided by Professor Jong Dai Kim.

### 4.3. Qualitative Analysis of AME

HPLC was conducted in order to qualitatively analyze the AME according to a previously described method with minor modifications. Briefly, the test sample (AME) and the standard sample (phenolic compounds: caffeic acid, catechin, chlorogenic acid, *p*-coumaric acid, ferulic acid, hesperidin, naringin, quercetin, rutin, vanillic acid; Sigma-Aldrich Co., St. Louis, MO, USA) were dissolved in 100% dimethyl sulfoxide (DMSO) and 5 mg/mL of stock solution was prepared, respectively. Using Waters 2695 Separation Module HPLC System (Waters Co., Milford, MA, USA) and Sunfire™ C_18_ column (inner diameter, 4.6 mm; length, 250 mm; Waters Co., Milford, MA, USA) filled with octadecylsilyl silica gel (diameter, 5 μm), 10 μL of test and standard samples were, respectively, chromatographed at 40 °C and 1.0 mL/min of flow rate. A (acetonitrile) and B (phosphoric acid, H_3_PO_4_) solutions were set as mobile phases under concentration gradient as follows: 0–23 min (A, 8; B, 92), 23–26 min (A, 15%; B, 85%), 26–36 (A, 30%; B, 70%), 36–40 min (A, 45%; B, 55%), 40–43 min (A, 45%; B, 55%), 43–45 min (A, 8%; B, 92%) and 45–53 min (A, 8%; B, 92%). The ingredients of AME were detected via using a Waters 996 Photodiode Array Detector (wavelength, 280 nm; Waters Co., Milford, MA, USA).

### 4.4. Experimental Groups and AME Treatment

The mice used in this study were assigned to three groups (n = 10 in each group) following shaving their dorsal hair: 1) non-UVB group, which was unexposed to UVB and treated with a vehicle (a mixture of propylene, ethanol and distilled water; combination ratio, 5:3:2), 2) UVB-vehicle group, which was exposed to UVB and treated with vehicle, and 3) UVB-AME group, which was exposed to UVB and treated with 1% AME.

The irradiation of UVB and treatment of AME were performed according to a published method [2]. In short, the mice were irradiated with a single exposure of UVB for 10 min using UVM-225D Mineralight UV Display Lamp (UVP, Phoenix, AZ, USA). The strength of the UVB beam was 150 mJ/cm^2^.

In the UVB-vehicle and UVB-AME groups, 200 μL vehicle or AME was applied to the dorsal skin once a day for 1 week.

### 4.5. Observation of CSS

As we previously described, we observed the clinical severity of skin injury induced by UVB irradiation by naked eyes and the diagnostic criteria was established according to measurement of severity of human dermatitis [2,38]. Briefly, we evaluated the clinical severity of the mice dorsal skin at 1, 3, 5 and 7 days after UVB irradiation. The skin conditions such as erythema/hemorrhage, scarring/dryness, edema and excoriation/erosion were scored as sum of the respective CSS score as follows: 0 (none), 1 (mild), 2 (moderate) and 3 (severe).

### 4.6. Tissue Preparation for Histological Analysis

Tissue processing was performed in order to perform a histological examination, as we previously described [39,40]. In short, the animals were administered with an intraperitoneal injection of 60 mg/kg of pentobarbital sodium (JW pharm. Co., Ltd., Seoul, Republic of Korea) for deep anesthetization [41]. Next, they were transcardially perfused into 3.5 mL/min of flow rate with 0.1 M phosphate-buffered saline (PBS, 0.85% NaCl *w*/*v*, pH 7.4) followed by 4% paraformaldehyde solution (in 0.1 M phosphate buffer, pH 7.4). Subsequently, their dorsal skin tissues were collected and fixed with the same solution for 6 h at room temperature. Afterwards, the tissues were embedded in paraffin blocks and, using a microtome (Leica, Wezlar, Germany), sectioned into 8 μm thickness.

### 4.7. H and E Staining

To investigate alterations in epidermal thickness and pathology, H and E staining was carried out in accordance with a precedent method [2,42]. In brief, the hippocampal sections were successively stained with H and E. The stained sections were dehydrated by a serial ethanol and mounted with Canada balsam (Kanto Chemical, Tokyo, Japan).

### 4.8. Masson’s Trichrome Staining

We conducted Masson’s trichrome staining in order to examine changes in collagen fibers of dorsal skin as we described previously [2,40]. Briefly, the sections were immersed with Biebrich scarlet-acid fuchsin solution (Sigma-Aldrich, St. Louis, MO, USA) and reacted with aniline blue (Sigma-Aldrich, St. Louis, MO, USA). Afterwards, the sections were dehydrated and mounted by cover glass using the same mounting medium.

### 4.9. Immunohistochemistry

According to our previously published method, we performed immunohistochemistry for collagens and MMPs. In short, sections were reacted with 0.3% hydrogen peroxide (H_2_O_2_) solution in 0.01 M PBS, pH 7.4 and, subsequently, immersed into 5% normal donkey or goat serum (in 0.01 M PBS, pH 7.4; Vector Laboratories, Inc., Burlingame, CA, USA) at room temperature for 30 min, respectively. Next, the sections were immunoreacted with each primary antibody overnight at room temperature: rabbit anti-collagen I (diluted 1:500; Abcam, Cambridge, UK), mouse anti-collagen III (1:500; Abcam, Cambridge, UK), rabbit anti-MMP-1 (diluted 1:250; Abcam, Cambridge, UK) and mouse anti-MMP-3 (diluted 1:250; Abcam, Cambridge, UK). Thereafter, the sections were reacted with corresponding secondary antibodies, which were conjugated with biotin for 2 h at room temperature: donkey anti-rabbit immunoglobulin G (IgG) (diluted 1:250; Vector Laboratories, Inc., Burlingame, CA, USA) and goat anti-mouse IgG (diluted 1:250; Vector Laboratories, Inc., Burlingame, CA, USA). These immunoreacted tissues were exposed to avidin-biotin complex (ABC, 1:300; Vector Laboratories, Inc., Burlingame, CA, USA) for 1 h at room temperature. The sections were finally visualized via reacting with 3,3′-diaminobenzidine tetrahydrochloride (DAB, in 0.01 M PBS; Sigma-Aldrich, St. Louis, MO, USA).

### 4.10. Data Analysis

Data were analyzed according to the previous studies [2,42]. In brief, the sections stained by H and E or Masson’s trichrome were monitored, respectively, via an AxioM1 light microscope (20X primary magnification; Carl Zeiss, Oberkochen, Germany) and taken by a digital camera (DP72, Olympus, Tokyo, Japan) linked to a personal computer (PC) monitor. Using Adobe Photoshop (version 8.0, Adobe, CA, USA) or using an image analyzing software (Optimas version 6.5; CyberMetrics, Phoenix, AZ, USA), the epidermal thickness or the intensity of fibrosis progress of dorsal skin, which is stained by Beibrich scarlet-acid fuchsin, was measured, respectively.

H-E stained fibroblast cells were counted into an array of 255 × 255 pixels corresponding to a tissue area of 50 × 50 μm. Each immunoreactive structure of collagens and MMPs were captured for quantitative analysis via using an AxioM1 light microscope (20× primary magnification; Carl Zeiss, Oberkochen, Germany), which is equipped with a digital camera (DP72, Olympus, Tokyo, Japan) connected to a PC monitor. The captured images were calibrated into an array of 512 × 512 pixels, which correspond to a tissue area of 140 μm^2^. Each immunoreactive structure was quantified by a 0 to 255 gray scale systems and using Adobe Photoshop (version 8.0, Adobe, CA, USA); a ratio of the relative optical density (ROD) of individual immunoreactivity for collagens and MMPs was calibrated as % that the non-UVB group was considered as 100%. The RODs were analyzed using NIH Image software (version 1.59; National Institutes of Health, MD, USA).

### 4.11. Statistical Analysis

In accordance with our previous study, the present data were shown as mean ± standard errors of mean (SEM) [2]. We applied one-way or multiple-way analysis of variance (ANOVA) and Tukey’s multiple range tests as a post hoc test using the criterion of the least statistically significant differences in order to test the differences among the experimental groups, using GraphPad Prism 5.01 software (GraphPad Software, Inc., La Jolla, CA, USA). We indicated statistical significance as under 0.05 of *p* value.
